# Prediction of bacterial small RNAs in the RsmA (CsrA) and ToxT pathways: a machine learning approach

**DOI:** 10.1186/s12864-017-4057-z

**Published:** 2017-08-22

**Authors:** Carl Tony Fakhry, Prajna Kulkarni, Ping Chen, Rahul Kulkarni, Kourosh Zarringhalam

**Affiliations:** 1 Department of Computer Science, University of Massachusetts Boston, 100 Morrissey Boulevard, Boston, 02125 MA USA; 2 Department of Physics, University of Massachusetts Boston, 100 Morrissey Boulevard, Boston, 02125 MA USA; 3 Department of Engineering, University of Massachusetts Boston, 100 Morrissey Boulevard, Boston, 02125 MA USA; 4 Department of Mathematics, University of Massachusetts Boston, 100 Morrissey Boulevard, Boston, 02125 MA USA

**Keywords:** CsrA, RsmA, Bacterial small RNA, ToxT, Boltzmann, RNA structure, Machine learning

## Abstract

**Background:**

Small RNAs (sRNAs) constitute an important class of post-transcriptional regulators that control critical cellular processes in bacteria. Recent research using high-throughput transcriptomic approaches has led to a dramatic increase in the discovery of bacterial sRNAs. However, it is generally believed that the currently identified sRNAs constitute a limited subset of the bacterial sRNA repertoire. In several cases, sRNAs belonging to a specific class are already known and the challenge is to identify additional sRNAs belonging to the same class. In such cases, machine-learning approaches can be used to predict novel sRNAs in a given class.

**Methods:**

In this work, we develop novel bioinformatics approaches that integrate sequence and structure-based features to train machine-learning models for the discovery of bacterial sRNAs. We show that features derived from recurrent structural motifs in the ensemble of low energy secondary structures can distinguish the RNA classes with high accuracy.

**Results:**

We apply this approach to predict new members in two broad classes of bacterial small RNAs: 1) sRNAs that bind to the RNA-binding protein RsmA/CsrA in diverse bacterial species and 2) sRNAs regulated by the master regulator of virulence, ToxT, in *Vibrio cholerae*.

**Conclusion:**

The involvement of sRNAs in bacterial adaptation to changing environments is an increasingly recurring theme in current research in microbiology. It is likely that future research, combining experimental and computational approaches, will discover many more examples of sRNAs as components of critical regulatory pathways in bacteria. We have developed a novel approach for prediction of small RNA regulators in important bacterial pathways. This approach can be applied to specific classes of sRNAs for which several members have been identified and the challenge is to identify additional sRNAs.

## Background

Bacterial survival in fluctuating environments requires an ability to make rapid adjustments to cellular gene expression. A key component of such adjustments to cellular phenotypes is post-transcriptional regulation. The stability of transcribed mRNAs and their protein production rates can be modulated by binding to non-coding regulatory RNA molecules called small RNAs (sRNAs) [1]. Many critical cellular processes, e.g. bacterial quorum-sensing, involve regulation by sRNAs as a central component [2]. Several reviews have highlighted the regulatory roles of bacterial sRNAs [3–5] and a major challenge for future work is the discovery of novel sRNAs and the elucidation of their regulatory functions.

Developments in high-throughput approaches such as RNA sequencing have led to unprecedented insights into bacterial transcriptomes. New classes of non-coding regulators have been discovered and several candidate sRNAs have been identified [6–10]. However, for a majority of the candidate transcripts, it remains to be elucidated whether these serve as functional sRNAs. Even for transcripts that have been analyzed further and established as *bona fide* sRNAs, the cellular regulatory functions are largely unknown. Furthermore, bacterial transcriptomes can vary significantly under different conditions suggesting that many condition-specific sRNAs have not yet been identified. Finally, it is likely that the sRNA repertoire remains largely unexplored for bacterial species for which high-throughput transcriptomic studies have not been carried out so far. There is thus a need for computational approaches that complement current experimental methods for the discovery and analysis of bacterial sRNAs.

Several computational methods and bioinformatics tools have been developed to enable genome-wide predictions for sRNAs [11–15]. Some approaches are based on comparative sequences and the conservation of sRNAs across genomes. However many sRNAs are species-specific and not conserved across different closely-related genomes. In addition to sequence-based methods, approaches focusing on RNA structure have also been developed [14–17]. However, recent high-throughput studies have identified multiple sRNA candidates, which are not predicted by existing computational tools [18, 19], indicating the need for novel computational approaches.

One approach for discovery of sRNAs along with insights into their regulatory functions is to focus on specific classes of sRNAs that are part of well-studied pathways or regulons. For example, the RNA-binding protein RsmA (CsrA) is a global regulator of gene expression in diverse bacterial species (henceforth denoted as RsmA for notational simplicity) [20–22]. The activity of RsmA is known to be regulated by the expression of sRNAs [23–27], however there are several bacterial species with RsmA orthologs for which the corresponding RsmA-regulating sRNAs are not known. Another example comes from the regulon of the virulence master regulatory protein ToxT in *Vibrio cholerae*. ToxT is a regulatory protein that belongs to the AraC/XylS family of transcription factors [28]. While previous work had identified a regulatory small RNAs activated by ToxT [29], a recent transcriptomic approach has identified multiple new sRNAs that are regulated by ToxT [18]. The development of computational approaches that lead to predictions for new sRNA members of these regulons is thus an important step in the development of general approaches for the discovery of specific classes of bacterial small RNAs.

In this work, we develop a novel approach that combines sequence and structure-based features in combination with machine-learning approaches to predict specific classes of sRNAs in bacterial genomes. Our approach is based on 1) deriving a set of sequential and structural features that can distinguish a given specific class of RNAs from other RNAs and 2) increasing robustness of predictions and modeling variation in training data using an ensemble approach. In combination with tools to characterize binding sites for transcription factors, the bioinformatics approach developed can be used to predict candidate sRNAs that are part of well-studied pathways. Knowledge of the pathways involved provides insight into the potential regulatory roles of the predicted sRNAs.

To illustrate our approach, we focus on the RsmA pathway in multiple bacterial genomes as well as the ToxT pathway in *Vibrio cholerae* and make predictions for novel sRNAs in these pathways. For the RsmA pathway, we use the extensive set of known RsmA-regulating sRNAs for training our machine-learning algorithm, which is then used to discover new RsmA-regulating sRNAs on a genome-wide scale. For the ToxT pathway, the set of currently known ToxT-regulated sRNAs is limited. In this case, in addition to using the set of know sRNAs in *Vibrio cholerae* as the training set, we also characterize ToxT binding site sequences upstream of potential sRNAs to increase the confidence in the predictions. We have developed a web-interface for predicting sRNAs in the RsmA pathway available at http://markov.math.umb.edu/inveniresrna/ to make the predictions and the tools available to different groups. The proposed approach can be generalized and applied to diverse bacterial regulons and can potentially accelerate the discovery of regulatory small RNAs in such pathways. In addition to the webserver, and in order to facilitate extensions of our models to other classes of sRNAs, we provide an R package InvenireSRNA, available for download at http://github.com/carltonyfakhry/InvenireSRNA.

## Methods

### Overview of approach

RNA classes typically consist of RNAs with similar structure and function. Such RNA classes can often be categorized based on the sequence composition and structural characteristics of the RNA molecules. Indeed, clustering according to sequence-structure similarity has now become a generally accepted scheme for non-coding RNA annotation [30]. For instance, in bacterial sRNAs, specific sequential-structural motifs (such as the presence of a Rho-independent terminator at the 3 ^′^ end) have a higher probability of appearing in specific structural conformations in the ensemble of low free energy structures. Such sequence-structure based signatures can be used to train machine learning algorithms that can be applied on a genome-wide scale to identify putative RNAs in the given class. In the next section we describe a novel method for feature generation for any given class of RNAs.

### Feature generation

Let *r*_0_,*r*_1_,⋯,*r*_*n*_ represent the sequence of a given RNA transcript of length *n* where *r*_*i*_∈{*A*,*C*,*G*,*U*} for *i*=1,⋯,*n*. Based on the sequential and structural conformations of the RNA, we constructed a set of features as follows.

In a fixed given secondary structure in the ensemble of all possible structures, nucleotides are either paired or unpaired. Hence, we may view the structure of *r*_0_,*r*_1_,⋯,*r*_*n*_ as a binary sequence $S=\{s_{i}\}_{i = 0}^{n}$, with 1 indicating that the nucleotide is paired and 0 indicating that the nucleotide is unpaired. Consider any 3 adjacent nucleotides (triplets) in the RNA sequence. There are 8 possible structural conformation for the triplet, namely 000,001,⋯,111. On the other hand there are a total of 64 possible nucleotide triples (*A* *A* *A*,*A* *A* *C*,⋯,*U* *U* *U*). Combining all possible triples-structural possibilities, we obtain 512 possible sequence-structure combinations, (AAA, 000), (AAA, 001), ⋯, (UUU, 111). We refer to these as sequential-structural composition (SSC) triplets.

Next, we construct a feature vector called *Boltzmann Triplet Feature, BTF*, by computing the probabilities of SSC triplets in the ensemble of low energy conformations. For a given RNA transcript, McCaskill’s algorithm [31] computes the Boltzmann partition function $Z = \sum _{S} \exp (-E(S)/RT)$, where the summation is over all secondary structures *S* of the RNA sequence, *E*(*S*) is the Turner free energy of *S*, *R* is the universal gas constant and *T* is absolute temperature. For a given secondary structure *S*_0_, the probability of the structure is given by *P*(*S*_0_)= exp(−*E*(*S*_0_)/*R* *T*)/*Z*. Hence, the probability of a given SSC triplet is given by $\sum _{\text {SSC} \in S}P(S)$, where the summation is taken over the structures that contain the given SSC triplet. The BTF vector is composed of the corresponding probabilities for all the SSC triplets.

**Fig. 1:**
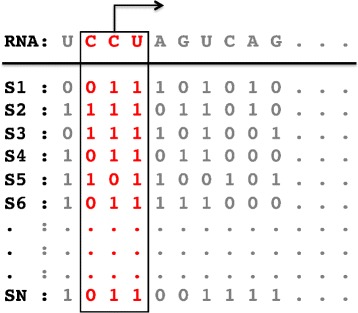
Schematic representation of BTF feature generation. For the given RNA sequence, a stochastic sample of low energy secondary structures is generated. Paired nucleotides are indicated by 1 and unpaired nucleotides are indicated by 0. For a given SSC triplet such as *ω*=(CCU, 011), the sequence and the sampled structures are scanned by sliding a window of length 3 over the sequence as well as samples and the frequency of *ω* is recorded

In order to estimate the probability of a given SSC triplet, we generate a stochastic sample of structures from the ensemble of low energy secondary structures consistent with the Boltzmann distribution. In our implementation, we used RNAsubopt program from the Vienna package to generate 1000 stochastic samples from the ensemble. We then track the number of times that the given SSC triplet (e.g, *ω*=(CCU, 011)) appears in the generated samples. That is, for each generated structure, we track the frequency of each SSC and compute the empirical probability of the SSC triplet over the randomly generated samples using a binomial model. Similar features have been used in classifying non-coding RNAs such as microRNAs [14, 15, 32]. If a given SSC triplet does not appear in samples, the corresponding probability is set to 0. Figure 1 shows a schematic representation of feature generation using our approach.

In addition to the BTFs described above, we consider some other features for classification of sRNAs. Specifically, we compute the probability of formation of a stem-loop at the 3 ^′^ end of the sequence by examining the occurrences of stem-loop in the stochastic samples. We also included a categoriacal feature indicating the presence or the absence of a Rho-independent terminator as defined in [33]. The definition of Rho-independent terminator includes the presence of a stem loop and a poly U tail at the 3^′^ end of the sequence plus a few more additional requirements [33].

### Construction of feature sets

To assess the ability of BTFs in classifying RNA classes, we generated the features for two specific classes of bacterial sRNAs, namely 1) RsmA regulating sRNAs in bacterial species with RsmA homolog and 2) sRNA targets of the master regulator ToxT in *Vibrio cholerae*. As will be described later, the features were used to train binary classifiers for predicting new sRNAs in each class.

#### Feature sets for RsmA regulating sRNAs

We obtained the “seed sequences” of RsmA-regulating sRNAs in bacterial species with known RsmA homologs as classified by Rfam [16]. There are a total of 105 seed sequences, including sRNAs that have been experimentally validated as regulators of RsmA. The features were generated for these sequences and used as positive examples for training. As is the case in many biological classification problems, one often has access to a set of positive examples; however, there is no well defined negative set. One commonly used approach for construction of a negative set, is to use a dinucleotide frequency preserving shuffle of the positive set [16, 17]. In our approach, we also constructed a negative set by shuffling the seed sequences while keeping the dinucleotide frequencies fixed using the Altschuldt-Erickson algorithm [34]. However, in addition to enforcing dinucleotide similarity between the negative and positive sequences, we examined the distribution of minimum free energies (MFE) of the positive sequences in order to produce a negative set that is structurally within a similar range. Positive sequence were shuffled multiple times and shuffled sequence with MFEs within the same range as positives were selected as the negative set.

For the test set, we obtained the sequences of predicted sRNA regulators of RsmA in all bacterial species with known RsmA homologs from Rfam. Note that the vast majority of the sequences in the test set are computationally predicted sRNA regulators of RsmA with no experimental support. However, we expect a large number of these sequences to be predicted as sRNA regulators of RsmA with our algorithm as well. There are a total of 1342 such sequences. We calculated the features for these sequences and the results were used in classification of the test set.

#### Feature sets for ToxT regulated sRNAs in *Vibrio cholerae*

As discussed before, to generate the training set we used all the previously annotated sRNAs of *Vibrio cholerae*. Since the size of the positive set is too small (total of 21 sequences) [19] for meaningful classification, we sought to expand this set by adding additional examples (total of 73 sequences) reported in a recent transcriptomic study performed in *Vibrio cholerae* [19].

From the additionally added sequences, we held out a total number of 4 sequences in order to construct a test set (resulting in a training set of size 90). In addition to these sequences, we considered 7 more sequences for the test set, obtained from another recent transcriptomic study in *Vibrio cholerae* [18]. The 11 test sequences were selected using the following filtering procedure. First, we scanned the genome of *Vibrio cholerae* for presence of a Rho-independent terminator downstream of the regions annotated as potential sRNAs in the studies. The software Arnold was used to carry out the search for terminators [35]. The sequences with no predicted Rho-independent terminators were filtered out from the test set. Next, we developed Position Weight Matrices (PWMs) for transcription factors in *Vibrio cholerae* using the RegPrecise database [36]. The PWMs were then used to scan the upstream regions of the remaining sRNAs for TF binding sites and the sRNAs with no binding site were further filtered out from the set. Using this approach, we identified the 11 potential sRNAs which show the presence of a Rho-independent terminator and an upstream binding site for a known transcription factor in *Vibrio cholerae*. This constituted the test set for sRNAs in *Vibrio cholerae*. This procedure was applied in order to increase the confidence that the test set sRNAs are *bona fide* sRNAs. The negative set was constructed in a similar manner as in RsmA.

#### Prediction sets for sRNAs in the RsmA and ToxT pathways

In order to predict new sRNA regulators of RsmA, we first obtained intergenic non-coding sequences using the Web server RSA Tools [37]. We then scanned the non-coding intergenic regions of selected bacterial genomes and identified regions containing 1) two or more ANGGA sequences (identified as RsmA binding motifs) followed by a poly U tail within 60 bp of the last ANGGA motif and 2) a Rho-independent terminator as predicted by the Arnold terminator prediction software [35]. The poly U tail constitutes the 3 ^′^ end of the putative sequences. For the 5 ^′^ end we used a variable window with different lengths, with the range motivated by examining the length distribution (specifically the distance of the first ANGGA motif from the known 5 ^′^ end) of positive sRNA sequences.

We constructed a putative set for potential new ToxT sRNA targets as follows. Previous studies focusing on DNA binding and regulation of target genes by ToxT have shown that ToxT can bind as a monomer to a 13-bp sequence designated as a toxbox sequence [38]. However all known ToxT target genes, with the exception of *aldA* [39], have been shown to have 2 upstream toxbox sequences in close proximity, suggesting that interaction between ToxT monomers is important for ToxT-dependent activation. Systematic mutagenesis studies for the binding sites have uncovered several key requirements for the toxbox sequences to ensure ToxT-based regulation [38].

The sequence requirements for toxbox sequences elucidated by previous binding and mutagenesis studies were combined to generate search criteria for upstream regions of genes regulated by ToxT. We considered all possible orientations of the two toxbox sequences (e.g. direct/inverted repeat) and also allowed the spacer region between the genes to be between 2-12 nucleotides. Using available information regarding known binding sites, we generated a Position Weight Matrix (PWM) representation for both toxbox1 and toxbox2 binding sequences. Specifically, toxbox sequences upstream of validated targets were categorized as toxbox1 or toxbox2 (based on specific sequence constraints identified in previous work). The frequency of occurrence of each nucleotide at a given position in the binding site was used to define the corresponding PSWM for each of the toxbox sites. The corresponding PSWMs were then used to identify putative toxbox sequences upstream of sRNA genes in the *Vibrio cholerae* genome. Next we scanned the noncoding intergenic regions of the genome for presence of two ToxT binding sites using the derived PWMs. We required that the ToxT binding sites should have a separation of 3≤*n*≤13. Moreover, we further examined the region for presence of a poly U tail starting from 12 bp downstream of the binding sites. For the 5 ^′^ end of the putative sRNA various window sizes were used.

### Classification

We employed a combined *ensemble-bootstrap* approach to in order to enhance classification robustness to multiple sources of variability and to increase the reproducibility of the models. As discussed in Methods, negative sequences were generated by random shuffles of positive sequences, while preserving the dinucleotide frequencies and the range of minimum free energies of secondary structures. Such negative sets are inherently variable. This variability must be taken into consideration to improve the reproducibility of the predictions. As such, we took an ensemble approach for training a series of binary classifiers, each trained on positive sequences and a different randomly generated negative set. In our implementation, we generated 100 negative data sets. For classifier, we used *L*_1_-regularized logistic regression [40].

*L*_1_ regularization automatically selects the most predictive features among all possible features. Automatic selection of predictive features depends on a tuning parameter *λ*, which in turn is optimally selected by cross validation. Due to randomness in cross validation folds, there will be slight variations in the selected predictive features. Additionally, variation in positive sequences will also impact the choice of the predictors by *L*_1_ regularization. For instance, if more positive sequences are added or removed from the model, some variation is expected in the selected model. To further increase the robustness of the classification to these sources of variation, we performed a bootstrap analysis during model training as follows.

For each training data, we first performed a bootstrap analysis by generating a total of 1000 bootstrap samples from the data. In each sample, an equal number of negative and positive examples were randomly selected from the data to fit the model. A classifier was trained separately using each sample and the total number of times that each feature was selected as significant was recorded. The features that were not always set to zero during the bootstrap process by the *L*_1_ penalty were then used as robust features. An *L*_1_-regularized logistic regression was subsequently fitted on the final set of robust features. This process was repeated for each of the training datasets (total of 100, one per each negative set). This results in an ensemble of 100 trained classifiers.

The performance of the trained models were assessed by cross validation as well as performing predictions on independent test sets. Features were generated for each test sequence and the set of 100 trained models were used to make a prediction on each new sequence. The final class label was decided by averaging over all model predictions. For cross validation, 10 negative sets were used due to speed limitations. In our implementation, we utilized a 10-fold cross validation.

Predictions on putative sequences were performed in a similar manner as in independent test sequences using the 100 trained classifiers. The model performance results and the new biological findings are presented in Results.

### Webserver

We provide an R Shiny based web-server that performs predictions on putative RsmA regulating sRNAs:“http://markov.math.umb.edu/inveniresrna/".

### R package

The source code and an R package InvenireSRNA is provided at: “http://github.com/carltonyfakhry/InvenireSRNA”. The package provides various functionalities, including extensions for training new classes of RNAs.

## Results

### Model Validation

**Fig. 2:**
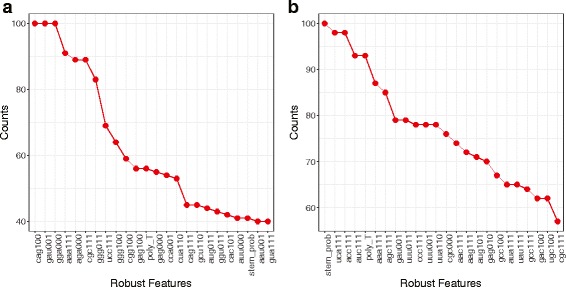
Features. Counts for Robust Features in classifier training for sRNAs in **a** RsmA pathway and **b** ToxT pathway

We performed several tests to assess the accuracy of the models in predicting sRNAs. As mentioned in the previous section, 100 training sets were generated by varying the negative sequences. For each training set, *L*_1_ regularized logistic regression classifiers were trained on 1000 bootstrap samples from the training set. Robust features were identified by tracking the frequency of the number of times that each feature was picked by the classifier across the bootstrap samples and training sets. Figure 2 shows the selected top features.

Figure 2 a indicates that the presence of a GGA motif in a single-stranded region is a strong predictor of RsmA regulating sRNAs, in agreement with experiments [41, 42]. Moreover, previous experimental work has shown that RsmA can bind to an AGAGA motif on mRNA leader sequences [43]. In agreement with this, our analysis indicates that having a AGA motif as well as having GAG motif in single-stranded regions serves as useful predictors for RsmA-binding sRNAs. Other important features include the presence of a poly U tail at the 3 ^′^ end, which is an indicative characteristics of RsmA regulating sRNAs. In case of sRNA targets of ToxT, existence of Rho-independent terminator (stem_prob) and a poly U tail at the 3 ^′^ end are among the strongest predictors (Fig. 2 b).

To assess the predictive power of the robust features in classifying sRNAs, we performed a 10-fold cross validation on 10 separate datasets. Table 1 shows the cross validation results. As can be seen, the models perform very well in terms of correctly classifying the sRNAs in their respective classes.

**Table 1:** Cross validation results for sRNA classifier

Class	Sensitivity	Specificity	Accuracy	Precision	AUC
RsmA	0.99	1	1	1	1
ToxT	0.91	0.93	0.92	0.93	0.99

First row: RsmA regulating sRNAs; Second row: sRNA targets of ToxT

Finally we tested the ability of the trained models in predicting sRNAs using independent test sets. In the case of RsmA regulating sRNAs, 1325 out 1342 (∼98.7*%*) were predicted as sRNAs by our method. Note that the test set in this case contains computationally predicted sRNA regulators by RsmA with no experimental support. In the case of sRNA targets of ToxT, 7 out of the 11 high confidence (probability>0.85) sequences were predicted as sRNAs by our method.

### Predictions of novel RsmA-regulating sRNAs

The application of our approach to RsmA-regulating sRNAs leads to several novel predictions which supplement results obtained from our previous approach focusing on sequence alone [12]. This includes predictions for such sRNAs in Gram negative species such as *Geobacter sulferreducens* for which no RsmA-regulating sRNAs have been experimentally discovered to date. In species such as *Shigella flexneri* and *Acinetobacter ADP1* for which RsmA-regulating sRNAs have been discovered [44] or predicted [12] in previous work, our approach leads to predictions of additional RsmA-regulating sRNAs. Furthermore, it is noteworthy that so far, no RsmA-regulating sRNAs have been experimentally validated in Gram-positive bacteria. Our approach leads to predictions for such sRNAs in Gram-positive species such as *Oceanobacillus iheyensis*.

Having obtained predictions for RsmA-regulating sRNAs in a given species, we also carried out homology searches for the sRNA sequences within that species using nucleotide BLAST. This was done to identify additional putative sRNAs which were not included in the original prediction set since they did not satisfy the criteria imposed (such as presence of a Rho-independent terminator as determined by Arnold). Additional sRNA candidates thus identified were then analyzed using the classifier developed. Table 2 provides a list of species we analyzed along with the corresponding top predictions for RsmA-regulating sRNAs.

**Table 2:** Predictions of RsmA regulating sRNAs in selected bacterial species

Organism	Flanking genes	Orientation	Predicted 5^′^ end	Predicted 3^′^ end	Probability
Acinetobacter ADP1	ACIAD0018/ACIAD0019	→←←	25035	24917	0.99
	ACIAD2750/ACIAD2751	←→←	2690560	2690698	0.99
Geobacter sulfurreducens	KN400_0047/KN400_0048	←→←	55576	55646	0.93
	KN400_1076/phoR	←→←	1156573	1156660	0.99
	KN400_2615	Antisense	2843134	2843215	0.91
Oceanobacillus iheyensis	OB3267/OB3268	←→←	3404835	3404912	0.95
Pseudomonas putida KT2400	PP_1864/PP_1865	→→→	2085406	2085577	0.96
	PP_1865/PP_1866	→←←	2087405	2087227	0.96
	PP_1865/PP_1866	→→←	2087652	2087827	0.85
	asd/PP_1990	→→→	2256149	2256329	0.97
	PP_2113/PP2114	→→→	2412827	2413009	0.84
	PP_2114/PP_2115	→←→	2414845	2414666	0.91
	PP_2218/PP_2219	→←→	2530804	2530622	0.93
	PP_3547/PP_3548	←→←	4022257	4022439	0.95
	PP_3547/PP_3548	←←←	4022850	4022673	0.95
Pseudomonas syringae pv. tomato DC3000	PSPTO_1719/PSPTO_1720	→→←	1889433	1889570	0.83
	uvrB/PSPTO_2165	→→←	2380918	2381056	0.85
	PSPTO_2585/amt-2	→→→	2856216	2856355	0.97
	PSPTO_3273/PSPTO_3274	→←←	3699381	3699244	0.95
	PSPTO_3490/PSPTO_3491	←←→	3941102	3940967	0.77
	PSPTO_3490/PSPTO_3491	←←→	3941740	3941605	0.93
	PSPTO_3491	Antisense	3942111	3941974	0.93
	fadB/PSPTO_3518	←←→	3970534	3970396	0.97
	gcd/PSPTO_4197	→←←	4728863	4728726	0.94
	PSPTO_5182/PSPTO_5183	→←→	5898180	5898085	0.96
Shigella flexneri	S2642/S2643	→→→	2532358	2532472	0.93
Vibrio fischeri ES114	hemB/gpp	→←←	61386	61312	0.83
	pgi/cheX	→←←	315197	315113	0.91
	rpsO/pnp	→←→	525909	525838	0.96
	ydaL/copG	←←←	852728	852653	0.99
	VF_1096/VF_1097	→←←	1212030	1211908	0.99

Arrows indicate the orientations of the predicted sRNA (center) and the two flanking genes

It is noteworthy that all the species for which RsmA-regulating sRNAs have been experimentally validated have orthologs of the GacA/S two-component system, which is involved in the activation of the sRNAs. However, there are several species which have orthologs of the RsmA but do not possess any orthologs of the Gac system. For these species, our approach leads to predictions for RsmA-regulating sRNAs (provided in Table 2) indicating that sRNAs that regulate RsmA can be activated by other transcription factors. This observation suggests that even in bacterial species for which RsmA-regulating sRNAs have been discovered, there are likely to be additional sRNAs that are activated by systems distinct from the GacA/S system. The application of our approach to the *Pseudomonads* predicts that this is indeed the case, as discussed below.

**Fig. 3:**
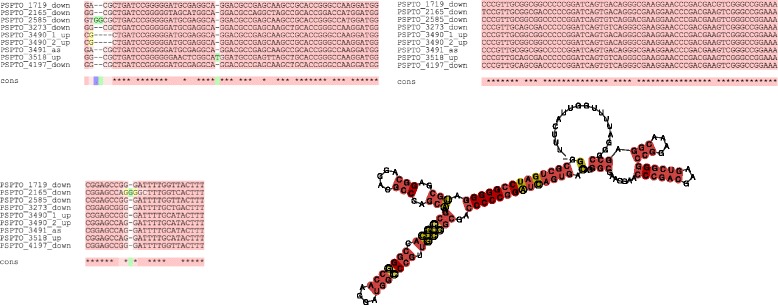
Structural conservation of RsmA-regulating sRNAs in *Pseudomonas syringae* as predicted by RNAz program. As can be seen, the predicted sRNA is highly conserved at the structural level, indicating that the predicted sRNA is functional. Note the presence of the GGA motif in the unpaired region of the predicted secondary structure

In *Pseudomonas syringae*, our approach predicts multiple novel RsmA-regulating sRNAs. It is interesting to note that these sRNAs show significant conservation at the sequence level (See Fig. 3). We analyzed the predicted sRNA sequences using RNAz software [45], which combines comparative sequence and structure prediction. The results (Fig. 3) show high Structural Conservation Index (*S* *C* *I*:0.69) indicating strong conservation at the level of secondary structure, and high RNA class probability (0.9), suggesting that the predicted sRNA is indeed functional. Furthermore, an analysis of the upstream regions of these predicted sRNAs reveals a conserved upstream site which is similar to the consensus *σ*_54_ (RpoN) binding site, suggesting that these sRNAs are activated by RpoN. Since RpoN is known to be a master regulator of virulence in *Pseudomonad syringae* [46], these predictions suggest additional connections between virulence regulation and the RsmA pathways.

Recent work has shown that bacterial sRNAs can also be derived from the 3 ^′^ UTR regions of coding genes [47, 48]. Interestingly, we obtain strong predictions for similar 3 ^′^ UTR derived sRNAs in the marine bacterium *Vibrio fischeri*. The sequences for these sRNAs include repeats and the predicted secondary structure shows the presence of multiple loop or single-stranded regions containing the GGA motif, suggesting that these sRNAs bind to RsmA. The genomic locations and upstream coding genes for all the predicted sRNAs is provided in Table 2. It would be of interest to test these predictions experimentally, since, if validated, these would constitute the first examples of 3 ^′^ derived sRNAs that regulate RsmA.

### Predictions of novel ToxT-regulated sRNAs

The preceding section considered a class of sRNAs for which we have multiple experimentally validated examples across several species. However, in many cases, it is of interest to consider small RNAs that are specific to a particular bacterial species. For example, the master regulator of virulence ToxT is primarily found in the different strains of the bacterial species *Vibrio cholerae*. It has been established that sRNAs are an integral component of the virulence pathways regulated by ToxT and it is of interest to expand the currently known set of sRNAs that are part of the ToxT pathway in *Vibrio cholerae*. As in the case of RsmA-regulating sRNAs, the classification approach leads to multiple predictions of ToxT-regulated sRNAs in *Vibrio cholerae*. Table 3 presents the prediction results for novel sRNAs.

**Table 3:** Predictions of ToxT regulated sRNAs in *Vibrio cholerae*

Organism	Flanking genes	Orientation	Predicted 5^′^ end	Predicted 3^′^ end	Probability
Vibrio cholerae	VC_0312/VC_0313	→←→	323707	323584	0.97
Vibrio cholerae	VC0967	antisense	1031946	1032143	0.97
Vibrio cholerae	VC_1192/VC_1193	→→←	1266285	1266383	0.94
Vibrio cholerae	VC_0249	antisense	255195	255110	0.94
Vibrio cholerae	VC_0994/VC_0995	←←→	1061082	1060968	0.98
Vibrio cholerae	VC_1072/VC_1073	→→→	1139343	1139442	0.94

Arrows indicate the orientations of the predicted sRNA (center) and the two flanking genes

**Fig. 4:**
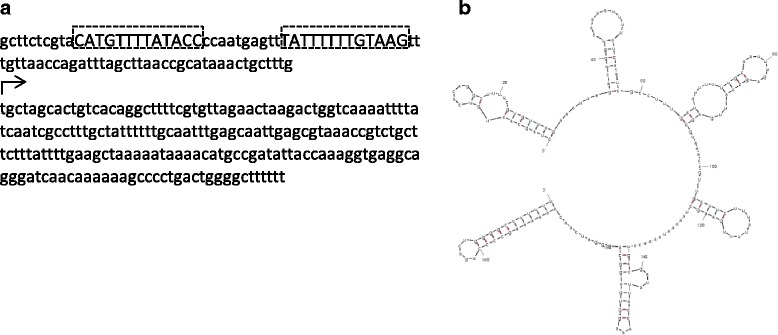
**a** Predicted sRNA and upstream sequence from VC0970 antisense region (flanking gene (in Table 3) is VC0967). The boxes indicate putative ToxT binding sites. The arrow indicates the start position of sRNA. **b** Predicted secondary structure of the sRNA using MFOLD

Figure 4 shows the sequence (with upstream ToxT binding site) and predicted secondary structure of the top-scoring prediction from our analysis. Our approach thus suggests that there may be several hitherto undiscovered sRNAs involved in the virulence of *Vibrio cholerae*. More generally, the approach developed can readily be replicated to consider sRNAs in other global regulatory pathways in *Vibrio cholerae* as well as other bacterial species.

## Discussion

A novel aspect of our approach stems from the generation and analysis of features that combine both sequence and structure-based information. Furthermore, we take multiple sources of variability into consideration to enhance the reproducibility of our predictions. For small RNAs regulating RNA-binding proteins, the results from the analysis provide insights for characterizing the corresponding protein binding sites. For example, the analysis for RsmA-regulating sRNAs indicates that having strong stem-loop regions and having the sequence motif GGA in single-stranded regions are important features of the RsmA binding site, as indeed has been demonstrated experimentally. Moreover, our analysis further indicates that having a GAG or AGA motif in single-stranded regions is an important component of the RsmA binding site on the sRNAs. Previous work has shown that RsmA can bind to an AGAGA motif on mRNA leader sequences [43], thus it would be of interest to experimentally validate if a GAG or AGA motif in single-stranded regions is important for RsmA binding as predicted by our work. More broadly, the analysis suggests that the approach used can lead to *de novo* discovery of motifs combining sequence and structure based information regarding binding sites for RNA-binding proteins.

Our previous approach for determining RsmA-binding small RNAs [12] focused primarily on sequence-based features such as the number of RsmA binding motifs and the presence of upstream binding sites for the transcriptional regulator GacA (which is an activator for the small RNAs in some bacterial species). This approach was able to make several new predictions for RsmA-regulating small RNAs, e.g. in species such as *Legionella pneumophila* which were validated by subsequent experiments. However this sequence-based approach is limited in species which do not have orthologs of the regulator GacA and in the discovery of small RNAs which have only a limited number of RsmA binding sites. In such cases, computational approaches need to take into account both sequence and structure-based features to identify potential RsmA-binding small RNAs and this requirement has been addressed in the novel approach developed in this work. Our current approach recovers all previously predicted small RNAs and also makes novel predictions for such small RNAs in several bacterial species including species which do not have orthologs of the transcriptional regulator GacA. Furthermore, we tested our approach by using as inputs the computational predictions for RsmA-regulating small RNAs available at RFAM [16]. The results indicate that ≈ 98.7% of the sRNAs annotated as RsmA-binding at RFAM are also predicted as RsmA-regulating sRNAs in our work. However, our approach also makes predictions for additional Rsma-reguilating sRNAs, some of which have been highlighted in Table 2. Finally, we note that our machine-learning approach using features which combine both sequence and structure-based information is quite general and can be used to predict novel members of any class of bacterial small RNAs. In particular, the code that has been developed also provides the user with the option of providing as inputs positive examples for any class of bacterial small RNAs. The code then calculates the features using the approach outlined and can be used to make predictions for any input candidate sRNA belonging to this class. Detailed instructions for applying our approach for general classes of bacterial sRNAs are provided and the package is available for download at: http://github.com/carltonyfakhry/InvenireSRNA.

The machine learning approach presented in this work makes several predictions which will be analyzed in detail, both experimentally and computationally, in future work. There are novel predictions for RsmA-regulating sRNAs in species where no sRNAs in this class have been discovered to date. There are also new predictions for sRNAs in species which already are known to have RsmA-regulating sRNAs. These predictions suggest that different environmental conditions or external stresses could activate different sets of sRNAs to control RsmA levels indicating that the set of RsmA-regulating sRNAs in bacteria is significantly larger than currently known. It would be of interest to validate these predictions experimentally in future work. The prediction of novel ToxT-regulated sRNAs in *Vibrio cholerae* would also be of interest to validate experimentally, given that the approach developed can readily be replicated to uncover sRNA components of pathways involving other master regulator proteins. It is hoped that the availability of these predictions through the Web tool and the R package that have been developed in this work will facilitate efforts in multiple labs to unravel regulation by specific classes of sRNAs in diverse species.

The involvement of sRNAs in bacterial adaptation to changing environments is an increasingly recurring theme in current research in microbiology. It is likely that future research, combining experimental and computational approaches, will discover many more examples of sRNAs as components of critical regulatory pathways in bacteria. In this work, we have developed a novel approach for prediction of bacterial sRNAs as components of specific regulatory pathways. While the present version makes several interesting predictions for current research, the approach developed can be generalized and applied more broadly. With the inclusion of additional features, the extension of this approach has the potential to open several new avenues of research. It would also be of interest to extend the current approach to focus on determining specific requirements for prediction of Hfq-binding sRNAs, a long-standing problem in the field. It is anticipated that further developments along these lines will lead to the discovery of novel sRNAs and an increased understanding of their role in cellular regulation.

## Conclusion

In summary, we have developed a machine-learning approach for prediction of small RNA regulators in important bacterial pathways. This approach can be applied to specific classes of sRNAs for which several members have been identified and the challenge is to identify additional sRNAs. We provide a web-interface for predicting sRNAs in the RsmA pathway available at http://markov.math.umb.edu/inveniresrna/. The application of our method leads to novel predictions for RsmA-regulating sRNAs in bacteria. The approach can also be applied to predict novel sRNAs regulated by specific transcription factors in a given bacterial species, as demonstrated in the case of the master regulator ToxT in *Vibrio cholerae*. The provided R package InvenireSRNA contains several functions that facilitate extension of our model to new classes of sRNAs.

## Abbreviations

BTF: Boltzmann triplet feature
CSrA: Carbon storage regulator A
RsmA: Repressor of secondary metabolites
sRNA: Small RNA
SSC: Sequential-structural composition
MFE: Minimum free energy
PWM: Position weight matrix
